# Complete Genome Sequence of Mycobacterium marinum Strain 050012 Isolated from Infected Skin Tissue

**DOI:** 10.1128/mra.00174-23

**Published:** 2023-05-01

**Authors:** Feifei Zhao, Yu Feng, Chengcheng Wang, Yi Xie, Dan Zhou, Yuling Xiao, Zhiyong Zong

**Affiliations:** a Center of Infectious Diseases, West China Hospital, Sichuan University, Chengdu, China; b Center for Pathogen Research, West China Hospital, Sichuan University, Chengdu, China; c Department of Laboratory Medicine, West China Hospital, Sichuan University, Chengdu, China; d Division of Infectious Diseases, State Key Laboratory of Biotherapy, Chengdu, China; Loyola University Chicago

## Abstract

We report the complete genome sequence of a Mycobacterium marinum strain, which was isolated from skin tissue of a wound infection. This strain was subjected to short- and long-read sequencing. Its complete genome contains a single 6,393,703-bp circular chromosome. Phylogenomic analysis of all M. marinum genomes assigned this strain to cluster I.

## ANNOUNCEMENT

Mycobacterium marinum is an opportunistic human pathogen (1) and is closely related to Mycobacterium ulcerans, Mycobacterium pseudoshottsii, and Mycobacterium shottsii (2–4). Here, we report the complete genome sequence of M. marinum strain 050012.

Infected skin tissue was excised from a local patient in 2022 and was homogenized in saline solution and then incubated on Lowenstein-Jensen medium at 37°C until colonies were visible. A single colony was subcultured on Lowenstein-Jensen medium at 37°C for 4 days for genomic DNA extraction. This study was approved by West China Hospital Ethics Committee, with informed consent being waived.

Genomic DNA was extracted using a QIAamp minikit. Large DNA fragments were recovered using a Blue Pippin recovery system. Barcodes were added using PCR-free EXP-NBD104, adapters were ligated using a SQK-LSK109 connection kit (Oxford Nanopore), and long-read sequencing was performed using the Nanopore PromethION platform. Raw Nanopore signals were subjected to base calling, demultiplexing, and adapter trimming using Guppy v6.3.9 (5) with a super-high-accuracy model (-c dna_r9.4.1_450bps_hac.cfg). Raw Nanopore reads (186,372; coverage, 292×; *N*_50_, 11,901 bp) were filtered using NanoFilt v2.8.0 (6). Reads with >1,000 bases and >8 average quality scores were assembled *de novo* using Canu v2.2 (7), Flye v2.9 (8), Miniasm v0.3 with Minipolish v0.1.3 (9), and Raven v.1.8.1 (10), separately, which produced a consensus assembly using Trycycler v0.5.3 (11), followed by three polishing rounds with Medaka v1.7.2 (https://github.com/nanoporetech/medaka).

The same DNA batch was fragmented by sonication, and 350-bp inserts were used to construct a library using a NEBNext Ultra kit, which was sequenced on a NovaSeq system (Illumina). Paired reads (6,897,636; 2 × 150 bp; coverage, 323×) were trimmed using Trimmomatic v0.39 (https://github.com/usadellab/Trimmomatic) and were used to polish the Nanopore assembly with two alternating rounds of Polypolish v0.5.0 (12) and POLCA from MaSuRCA v4.1.0 (13). Default parameters were used for all software unless otherwise specified.

The complete genome contains a 6,393,703-bp chromosome (G+C content, 65.78%) rotated to start with *dnaA* with 5,267 coding sequences and annotated using Prokka v1.14.6 (14). Average nucleotide identity (ANI) between 050012 and M. marinum CCUG20998^T^ (accession number CP024190.1), *M. pseudoshottsii* JCM15466^T^ (AP018410.1), *M. shottsii* JCM12657^T^ (AP022572.1), and M. ulcerans Agy99^T^ (CP000325.1) was 98.56%, 98.36%, 97.70%, and 98.09%, respectively, as determined using FastANI v1.33 (15). Although the ANI values agree with regard to their synonymy, the aforementioned species are regarded as different species on the basis of different clinical features, host spectra, and pathogenicity (16). Nevertheless, the characteristic multiple copies of IS*2404*/IS*2606* in *M. pseudoshottsii* and M. ulcerans and IS*Mysh01*/IS*Mysh03* specific to *M. shottsii* were absent from 050012 (3, 17, 18), confirming that it was M. marinum.

**FIG 1:**
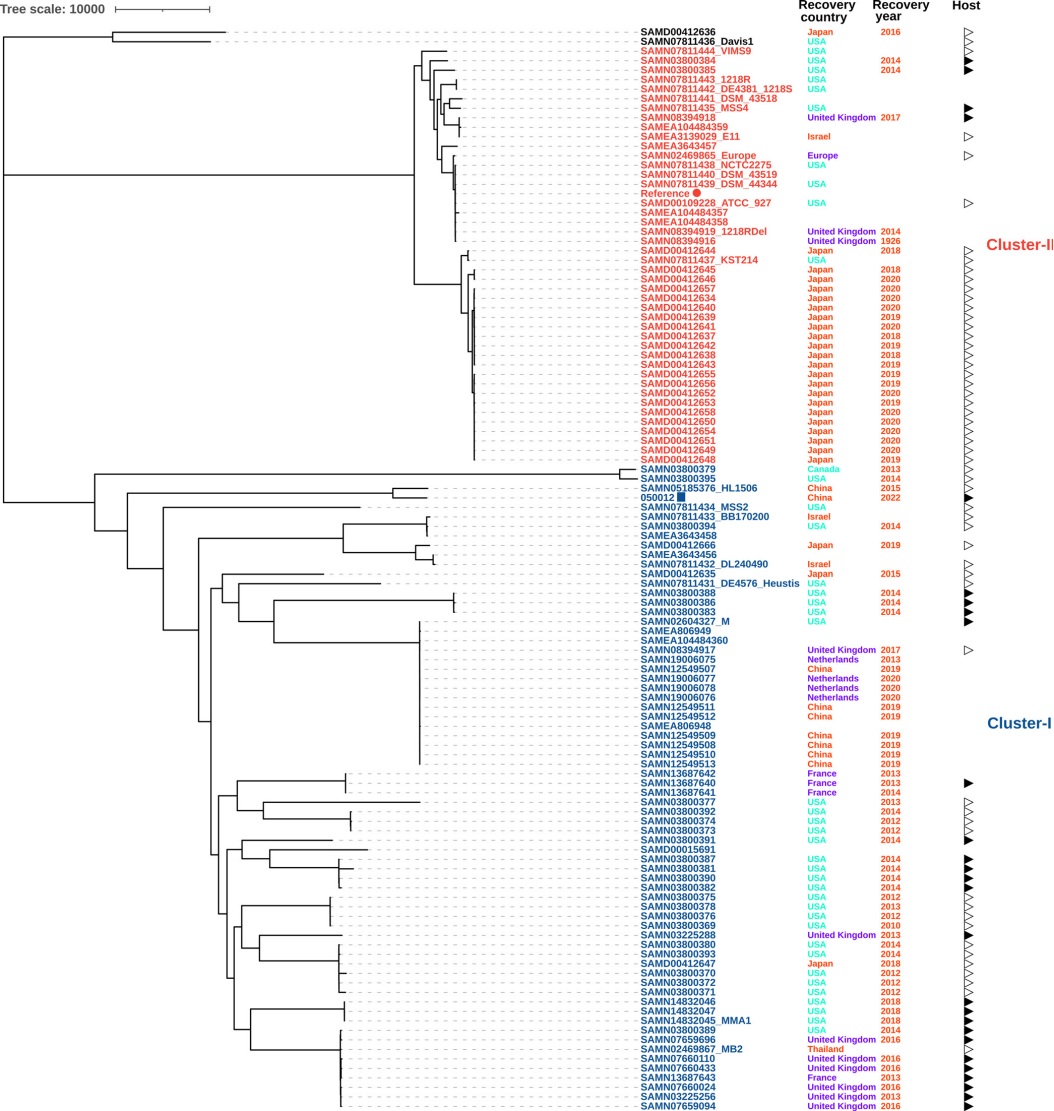
Phylogenomic tree of M. marinum. The tree was based on SNPs compared to the reference strain M. marinum CCUG20998^T^. The reference strain and strain 050012 in this study are indicated with a red circle and a blue rectangle, respectively. The country and year of recovery are shown. Human host and nonhuman sources such as fish and environment are indicated by filled and blank triangles, respectively. Strains belonging to the major clusters I (blue) and II (red) are marked in blue and red, respectively. The tree was visualized using iTOL (https://itol.embl.de/). The scale bar shows the number of nucleotide substitutions.

We retrieved all M. marinum genome assemblies (*n* = 51) and SRA reads (*n* = 88) from NCBI. SRA reads were trimmed using Trimmomatic and assembled *de novo* using SPAdes v3.15.3 (19). Duplicated genomes (*n* = 26) were removed. All genomes (*n* = 113) were identified as M. marinum as described above. Single nucleotide polymorphisms (SNPs) were called using M. marinum CCUG20998^T^ as a reference using Snippy v4.6.0 (https://github.com/tseemann/snippy) with --ctgs option and Gubbins v3.2.1 with a general time-reversible (GTR) model plus gamma distribution and a 100-bootstrap test (20). The phylogenomic tree shows that M. marinum strains belong to two major clusters (I and II), consistent with previous findings (21). 050012 and all strains from China belong to cluster I (Fig. 1). The 050012-containing branch also comprises strains from the United States and Europe. Further analyses of M. marinum genomes are warranted.

### Data availability.

Complete genome sequences of Mycobacterium marinum 050012 have been deposited in GenBank under BioProject accession number PRJNA929715, BioSample accession number SAMN32983127, SRA accession numbers SRR23450433 (Nanopore reads) and SRR23294010 (Illumina reads), and GenBank nucleotide accession number CP118910.
